# Cinnamic acid mitigates methotrexate-induced lung fibrosis in rats: comparative study with pirfenidone

**DOI:** 10.1007/s00210-023-02652-w

**Published:** 2023-08-15

**Authors:** Eman Abdalhameid, Enas A. Abd El-Haleim, Rania M. Abdelsalam, Gehan S. Georgy, Hala M. Fawzy, Sanaa A. Kenawy

**Affiliations:** 1 Department of Pharmacology and Toxicology, Egyptian Drug Authority (EDA), Giza, Egypt; 2 Department of Pharmacology and Toxicology, Faculty of Pharmacy, Cairo University, Cairo, Egypt; 3 Department of Biology, School of Pharmacy, Newgiza University, Giza, Egypt

**Keywords:** Methotrexate, Cinnamic acid, Pirfenidone, Lung fibrosis, Transforming growth factor-β, Collagen

## Abstract

**Purpose:**

Lung fibrosis is a heterogeneous lung condition characterized by excessive accumulation of scarred tissue, leading to lung architecture destruction and restricted ventilation. The current work was conducted to examine the probable shielding influence of cinnamic acid against lung fibrosis induced by methotrexate.

**Methods:**

Rats were pre-treated with oral administration of cinnamic acid (50 mg/kg/day) for 14 days, whereas methotrexate (14 mg/kg) was orally given on the 5^th^ and 12^th^ days of the experiment. Pirfenidone (50 mg/kg/day) was used as a standard drug. At the end of the experiment, oxidative parameters (malondialdehyde, myeloperoxidase, nitric oxide, and total glutathione) and inflammatory mediators (tumor necrosis factor-α and interleukin-8), as well as transforming growth factor-β and collagen content, as fibrosis indicators, were measured in lung tissue.

**Results:**

Our results revealed that cinnamic acid, as pirfenidone, effectively prevented the methotrexate-induced overt histopathological damage. This was associated with parallel improvements in oxidative, inflammatory, and fibrotic parameters measured. The outcomes of cinnamic acid administration were more or less the same as those of pirfenidone. In conclusion, pre-treatment with cinnamic acid protects against methotrexate-induced fibrosis, making it a promising prophylactic adjuvant therapy to methotrexate and protecting against its possible induction of lung fibrosis.

## Introduction

Methotrexate (MTX) has been widely prescribed in the treatment of a variety of malignant and inflammatory diseases for more than six decades and is still used for its remarkable therapeutic effects (Olsen et al. 2014; Dong et al. 2021). Furthermore, MTX possesses immunomodulatory properties due to its antimetabolite action, which interferes with folic acid metabolism. As MTX has a larger affinity for dihydrofolate reductase (DHFR) than folate, it inhibits the production of tetrahydrofolate reductase, which is essential for the biosynthesis of thymidine and purines, both of which are required for DNA synthesis (Rana et al. 2020; Chen et al. 2021). That is how it prevents cell division and protein production (Howard et al. 2016; Ghoneum and El-Gerbed 2021).

The initial instance of MTX-induced lung toxicity was documented in 1969 in children diagnosed with acute lymphoblastic leukemia (Jani et al. 2017). A significant proportion (60–93%) of individuals taking MTX medication encountered a range of clinical indications, such as difficulty breathing, coughing, elevated body temperature, breathing difficulties, pneumonia, lung inflammation, and fibrosis. Consequently, approximately 30% of these patients were compelled to halt MTX treatment as a consequence of these adverse reactions (Mammadov et al. 2019). A suggested mechanism for the development of lung toxicity caused by MTX involves the stimulation of pro-inflammatory cytokines such as tumor necrosis factor-α (TNF-α), interleukins (IL), and monocyte chemoattractant protein (MCP-1) release through the p38 mitogen-activated protein kinase (MAPK) pathway (Kalemci et al. 2018; Koppelmann et al. 2021). Excessive production of these cytokines, along with platelet-derived growth factor (PDGF) and transforming growth factor- β (TGF- β), leads to the proliferation and differentiation of fibroblasts to myofibroblasts, increasing extracellular matrix (ECM) proteins and collagen deposition, ultimately leading to fibrosis (Fikry et al. 2015; Chanda et al. 2019). Pulmonary fibrosis is a heterogeneous lung condition characterized by excessive accumulation of scar tissue, leading to the destruction of lung architecture and restricted ventilation. Under physiological conditions, fibrogenesis is initiated due to tissue injury to resolve the wound area through four stages, including: coagulation, inflammation, fibroblast proliferation, and remodeling phases, where the normal structure of tissue and its integrity are renovated (Kant et al. 2021). The first FDA-approved medicine for the treatment of idiopathic pulmonary fibrosis (IPF) was pirfenidone (Pir), although the mechanism by which it acts is not entirely known (Hadjicharalambous and Lindsay 2020; Wilfong and Aggarwal 2021). Pir possesses antioxidant, anti-inflammatory, and antifibrotic properties as it reduced the level of TGF-β, IL-1β, IL-6, Interferon-γ (IFN-γ), MCP-1, and other cytokines in the bleomycin model of lung fibrosis (Hadjicharalambous and Lindsay 2020; Shah et al. 2021).

Cinnamon (*Cinnamomum cassia*) has a wide range of uses as a herbal medicine (Liu et al. 2020). The main chemical compound found in cinnamon is cinnamic acid (cin), which is one of the most common and basic phenolic acids found in nature (Hong et al. 2021; Ben Lagha et al. 2021). Cin possesses a lot of beneficial pharmacological actions such as antitumor, antimicrobial, antioxidant, and anti-inflammatory activities (El-Sayed et al. 2013; Ruwizhi and Aderibigbe 2020). Consequently, hindering inflammation and oxidative stress was deliberated as a prospective beneficial goal in the treatment of fibrotic diseases. The existent study aims to assess the potential shielding effect of cin, correlated to Pir, in contravention of pulmonary fibrotic-associated MTX use in rats.

## Material

### Animals

Male Sprague–Dawley rats (200–250 g) obtained from the animal house of the National Organization for Drug Control and Research (NODCAR, Cairo, Egypt) were used. Animals were lodged for at least one week in the laboratory room before testing under standard housing conditions: room temperature (24–27°C), humidity (60 ± 10%), alternating 12 h light and dark cycles, free access to food (standard pellet diet), and water ad libitum.

The investigation was permitted by the ethics committee for animal experimentation of the college of pharmacy, Cairo University, Egypt (PT 1511; approval date: October 26, 2015) and followed the instructions for the care and use of laboratory animals published by the US National Institutes of Health (NIH Publication No. 85–23, revised 1996).

### Drugs and chemicals

- Dimethyl sulfoxide 50% (DMSO) was obtained from Sigma-Aldrich Corporation, Lyon, France, and was orally administrated in a dose of 5 ml/kg/day for 14 days.
- MTX injection (50 mg/2 ml) was obtained from Mylan pharmaceutical Inc, USA, was orally administrated to induce lung fibrosis in a dose 14 mg/kg once weekly for 2 consecutive weeks.
- Cin was provided from qualikems fine chemicals, New Delhi, Delhi, India, was orally given in a dose 50 mg/kg/day for 14 days**.** Cin was dissolved in 50% DMSO in concentration of (10 mg/ml).
- Pir was obtained from Cipla LTD, Rorathang, Sikkim, India, was orally administered as a standard drug in a dose 50 mg/ kg/day for 14 days. Pir was dissolved in saline in concentration of (10 mg/ml).

## Methods

### Experimental design

Adult male Sprague–Dawley rats (200–250 g) were randomly dispersed into seven groups of 5–8 rats each and treated as follows:

- Normal (saline) group: rats received saline orally daily.
- Normal (DMSO) group: rats received 50% DMSO (solvent for cinnamic acid) orally daily (El-Sayed et al. 2013).
- MTX group: rats received MTX, 14 mg/kg, orally once a week for 2 weeks; served as lung fibrotic group (Fikry et al. 2015).
- Pir group: rats were treated with pir (50 mg/kg/day) orally; serve as a standard drug control group (Song et al. 2018).
- Pir + MTX group: rats received pir (50 mg/kg/day) orally, whereas MTX (14 mg/kg) was orally given on the 5th and 12th days of the experiment; serve as a standard drug-treated group.
- Cin group: rats were treated with cin (50 mg/kg/day) orally (El-Sayed et al. 2013).
- Cin + MTX group: rats received cin in a dose of 50 mg/kg/day orally, whereas MTX (14 mg/kg) was orally given on the 5th and 12th days of the experiment.

The experiment lasted for 14 days; all solutions were given orally via a gavage needle.

At the finale, animals were decapitated, the lungs were erased, splashed with cold saline, blotted dry, and weighed. Three left lungs from different groups were conserved in 10% formalin for subsequent histopathological investigation. The used animals were frozen until they were incinerated.

- ***N.B.:*** There is no significant alteration between the normal (saline) group and the normal (DMSO) group results in the existing study.

### Preparation of lung homogenate

Lungs were homogenized in icy phosphate buffered saline, 1:10 w/v, and the homogenates were centrifuged at 13000 × g, 4°C, for 15 min. The supernatants were frozen at 80 °C in preparation for assessing the evaluated parameters.

### Evaluated parameters

#### Evaluation of oxidative stress biomarkers in lung homogenate

The lung myeloperoxidase (MPO) was estimated using a myeloperoxidase colorimetric activity assay kit provided by Sigma-Aldrich Co. The lung nitric oxide (NO) was estimated using the reagents of the rat NO ELISA kit provided by Abbkine, Inc., China.

The malondialdehyde (MDA) and total glutathione (tGSH) contents in the lung were estimated using the reagents of lipid peroxidation and total glutathione assay kits, respectively, provided by Eagle Biosciences Inc., Boston. All the measures were carried out as specified in the constructors’ directives.

#### Assessment of inflammatory markers in lung homogenate

The lung TNF-α and TGF-β contents were estimated utilizing the commercially available rat ELISA kit provided by Mybiosource, San Diego, USA. The IL-8 content was assessed by consuming the commercially accessible rat IL-8 ELISA Kit provided by Cloud-Clone Corp., USA. These assays employ the quantitative sandwich enzyme immunoassay technique as reported by the manufacturers’ directives.

#### Western blot analysis of collagen in lung homogenate

Collagen content in the lung was evaluated by Clarity™ Western ECL substrate provided by Bio-Rad Laboratories, Inc., Canada.

The ReadyPrep™ protein extraction kit (total protein) provided by Bio-Rad Inc. was employed and added to lung samples. (Catalog #163–2086) and Bradford Protein Assay Kit (SK3041) for quantitative protein analysis was provided by Bio Basic Inc. (Markham, Ontario, L3R 8T4 Canada). A Bradford assay was performed according to the manufacturer's guidelines to evaluate every sample's protein content. After that, an equal volume of 2 × Laemmli sample buffer containing 4% SDS, 10% 2-mercaptoehtanol, 20% glycerol, 0.004% bromophenol blue, and 0.125 M Tris HCl was added to each sample's 20 g protein concentration. The pH was measured and adjusted to 6.8. Before loading on polyacrylamide gel electrophoresis, each prior mixture was heated at 95 °C for 5 min to achieve protein denaturation.

##### Protein separation by electrophoresis

Samples were separated on a polyacrylamide gel; the procedure was shortened to SDS-PAGE (Sodium Dodecyl Sulfate PolyAcrylamide Gel Electrophoresis), which is a standard technique for separating proteins according to their molecular weight. Polyacrylamide gels were performed using the TGX Stain-Free™ FastCast™ Acrylamide Kit (Bio-Rad Laboratories, Inc.).

##### Protein blotting (transfer of proteins from the gel to the membrane) and blocking the membrane

The gel was arranged in a transfer sandwich as follows from underneath to on top: filtration paper, PVDF membrane, gel, and filtration paper. It was positioned in the transfer tank with 1 × transfer buffer, which consists of 25 mM Tris, 190 mM glycine, and 20% methanol. The blot was run for 7 min at 25 V to allow protein bands to transfer from the gel to the membrane using BioRad Trans-Blot Turbo. The membrane was blocked in tris-buffered saline with Tween 20 (TBST) buffer and 3% bovine serum albumin (BSA) at 37 °C for 1 h. The blocking buffer consists of the following ingredients: 20 mM Tris pH 7.5, 150 mM NaCl, 0.1% Tween 20, and 3% bovine serum albumin (BSA).

##### Incubation with the primary antibody

Primary antibodies for collagen 1 A1 (Catalog Number: sc-293182, Santa Cruz Biotechnology, Inc., Europe) were diluted in TBST according to factory-made directions. Incubation was done overnight at 4 °C, against the blotted target protein. The blot was washed with TBST for 3–5 rounds for 5 min. In the HRP-conjugated secondary antibody solution (goat anti-rabbit IgG-HRP-1 mg goat mab, Novus Biologicals), the blotted target protein was incubated for 1 h at 37 °C. TBST was used to wash the blot 3–5 times for 5 min.

##### Imaging and data analysis quantitation

The chemiluminescent substrate (Clarity™ Western ECL substrate, Bio-Rad) was applied to the blot according to the manufacturer's instructions. Solution A (Clarity Western luminal/enhancer solution) and solution B (peroxidase solution) were mixed in equal parts. A CCD camera-based imager was used to capture the chemiluminescent signals. On the ChemiDoc MP imager, image analysis software was used to read the band intensity of the target proteins against the control sample, ꞵ-actin (housekeeping protein), by protein normalization.

#### Histopathological examination

Lung samples from various groups were maintained in 10% Formol saline, for 24 h. After washing the samples in tap water, they were dehydrated using serial dilutions of alcohol (methyl, ethyl, and absolute ethyl). In a hot air oven, specimens were cleared in xylene and embedded in paraffin for 24 h at 56 ºC. A sled microtome was used to segment paraffin-beeswax tissue blocks at 4 µ thickness. Tissue sections were collected on glass slides, deparaffinized, and stained with hematoxylin and eosin, in addition to Masson trichrome stain, for inspection under a light electric microscope.

### Statistical analysis

Statistical analysis was achieved using instant automated software (Graph Pad Prism Software version 5.01, Inc., CA, USA). The results were implemented as the mean ± standard error of the mean (SEM). A one-way ANOVA followed by Tukey–Kramer multiple comparison tests was used. The results were deliberated significantly at P-value < 0.05.

## Results

### Evaluation of oxidative stress markers in lung homogenate

The present data displayed that MTX oral administration initiated a tremendous rise in oxidative stress, as shown by an upturn in MPO activity and NO lung content of 46% and 52%, respectively, as compared to the normal group. Similarly, an 8.6-fold intensification was detected in lung MDA levels after administration of MTX. Conversely, upon pre-treatment with cin, a 47% reduction in MPO activity was observed with a normalizing action in lung NO content and a noticeable drop in MDA content of 77%, related to the MTX control group. Using pir as a pre-treatment standard normalized MPO activity and significantly reduced the content of NO and MDA by 32% and 74%, respectively, relative to the MTX control group.

Meanwhile, a marked decrease in lung content of total GSH occurred in the MTX group by 66% in relation to normal rats. Upon pre-treatment with Cin or Pir, a significant elevation in the tGSH content by 5.4 and 3.4 folds, respectively, relative to the MTX control group was shown **(**Table 1).

**Table 1:** Evaluation of Oxidative stress markers in lung homogenate

Groups	MPO (milliunits/mg tissue)	NO (µmol/mg tissue)	MDA (µmol/mg tissue)	tGSH (µmol/mg tissue)
Normal	37.44 ^#^ ± 2.19	40.92 ^#^ ± 2.92	0.37 ^#^ ± 0.01 ^#^	287.40 ^#^ ± 24.13
MTX	82.30 ± 5.98	62.24 ^#^ ± 2.02	3.17 ± 0.02	97.80 ± 5.13
Pir control	39.28 ^#^ ± 2.56	39.06 ^#^ ± 1.09	0.38 ^#^ ± 0.01	306.00 ^#^ ± 20.94
Pir + MTX	37.94 ^#^ ± 2.76	42.26 ^#^ ± 2.38	0.82 ^* #^ ± 0.02	331.60 ^#^ ± 34.08
Cin control	38.36 ^#^ ± 1.77	42.50 ^#^ ± 2.27	0.39 ^# p^ ± 0.05	255.60 ^#^ ± 18.75
Cin + MTX	38.96 ^#^ ± 5.00	40.10 ^#^ ± 2.02	0.74 ^* #^ ± 0.05	528.00 ^* # p^ ± 24.14

Each value represents the mean of 5–8 rats ± SEM. Data were analyzed by One-way ANOVA followed by Tuckey-Kramer multiple comparison test. ^*^ *P* < 0.05 vs Normal group, ^#^ *P* < 0.05 vs MTX group, ^p^ *P* < 0.05 vs Pir + MTX

### Evaluation of inflammatory markers in lung homogenate

**Fig. 1:**
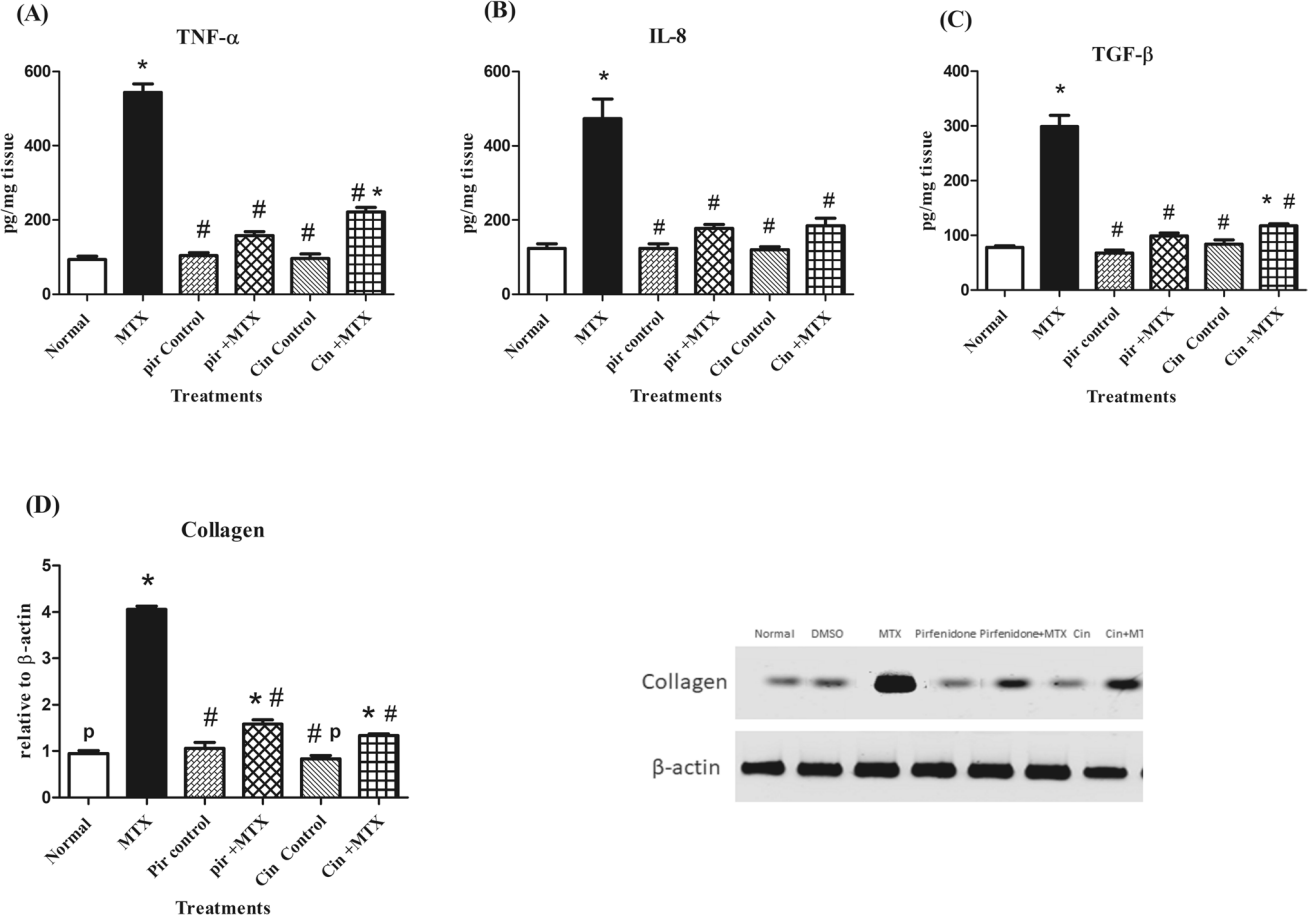
Evaluation of inflammatory markers and Western blot analysis of Collagen in lung homogenate. (**A**): Assessment of TNF-α content, (**B**): Assessment of IL-8 content, (**C**): Assessment of TGF-β content, (**D**): Western blot analysis of Collagen in lung homogenate. Each value represents the mean of 5–8 rats ± SEM. Data were analyzed by One-way ANOVA followed by Tuckey-Kramer multiple comparison test. * P < 0.05 vs Normal group, # P < 0.05 vs MTX group, p P < 0.05 vs Pir + MTX

Oral treatment with MTX exhibited a significant increment in lung **TNF-α** content by 5.4-fold in comparison with normal rats. Oral pre-treatment administration of Cin instigated a significant reduction in TNF-α lung content of 0.4-fold. Meanwhile, TNF-α content reduced significantly in Pir pretreated rats by 0.3-fold in association with the MTX group **(**Fig. 1A**).**

Rats receiving MTX displayed a significant augmentation in **IL-8** content by 3.8-fold compared to normal rats. Rats pretreated with cin and pir reduced lung content of IL-8 by approximately 62% compared to the MTX group (Fig. 1B).

Lung content of **TGF-β** was significantly increased following MTX administration by 3.8-fold as compared to the normal group. Conversely, a marked reduction in TGF-β content by 61% was presented in the cin-pretreated group. Likewise, the results revealed that oral administration of pir before MTX reduced the lung content of TGF-β by 67% relative to the MTX group (Fig. 1C).

### Western blot analysis of collagen in lung homogenate

A significant boost of the lung collagen content by 4.3-fold was obtained due to oral administration of MTX, linked to normal rats. However, a 67% and 61% depletion of the collagen content were detected in cin- and pir-pretreated rats, respectively, linked to the MTX control group (Fig. 1D).

### Histopathological examination

#### Hematoxylin and eosin (H&E) stain

**Fig. 2:**
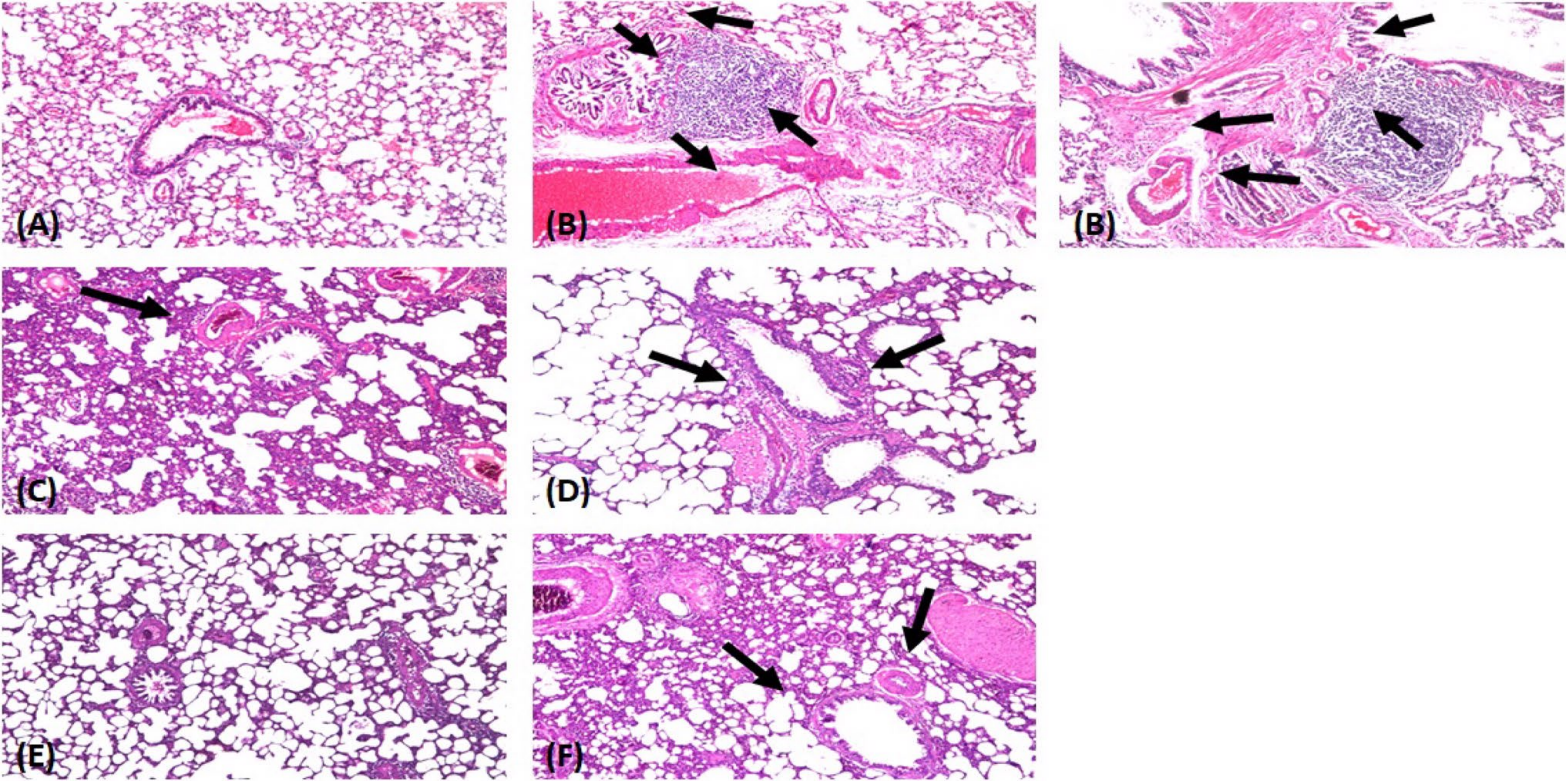
Photomicrographs of rat lung sections stained with H&E (X 16). (**A**) Normal group: normal histological structure of the bronchioles and surrounding air alveoli; (**B**) MTX treated group: there was fibrosis and collagen proliferation in the peribronchiolar area; (**C**) Pir treated group: few inflammation cells infiltration in perivascular tissue; (**D**) Pir + MTX treated group: few inflammation cells infiltration in peribronchiolar tissue; (**E**) Cin treated group: no histopathological alteration; (**F**) Cin + MTX treated group: sclerosis of the vascular wall

In the lung of the normal (saline) group, there was no histopathological alteration recorded (Fig. 2A).

Rats subjected to MTX showed peribronchiolar tissue and lymphoid follicle hyperplasia associated with dilatation in the blood vessels and inflammatory cell infiltration in the parenchyma (Fig. 2B). Treatment with cin alone showed a normal appearance of cells (Fig. 2E). However, the cin + MTX group exhibited thickening and hypertrophy of the vascular wall (Fig. 2F).

There was no histopathological alteration in the parenchyma, but the perivascular tissue presented few inflammatory cell infiltrations in the perivascular tissue of the pir control group (Fig. 2C). Moreover, the peribronchiolar tissue in rats subjected to MTX after pir treatment exhibited a thickened wall and few inflammatory cell infiltrations (Fig. 2D).

#### Masson’s trichrome stain

**Fig. 3:**
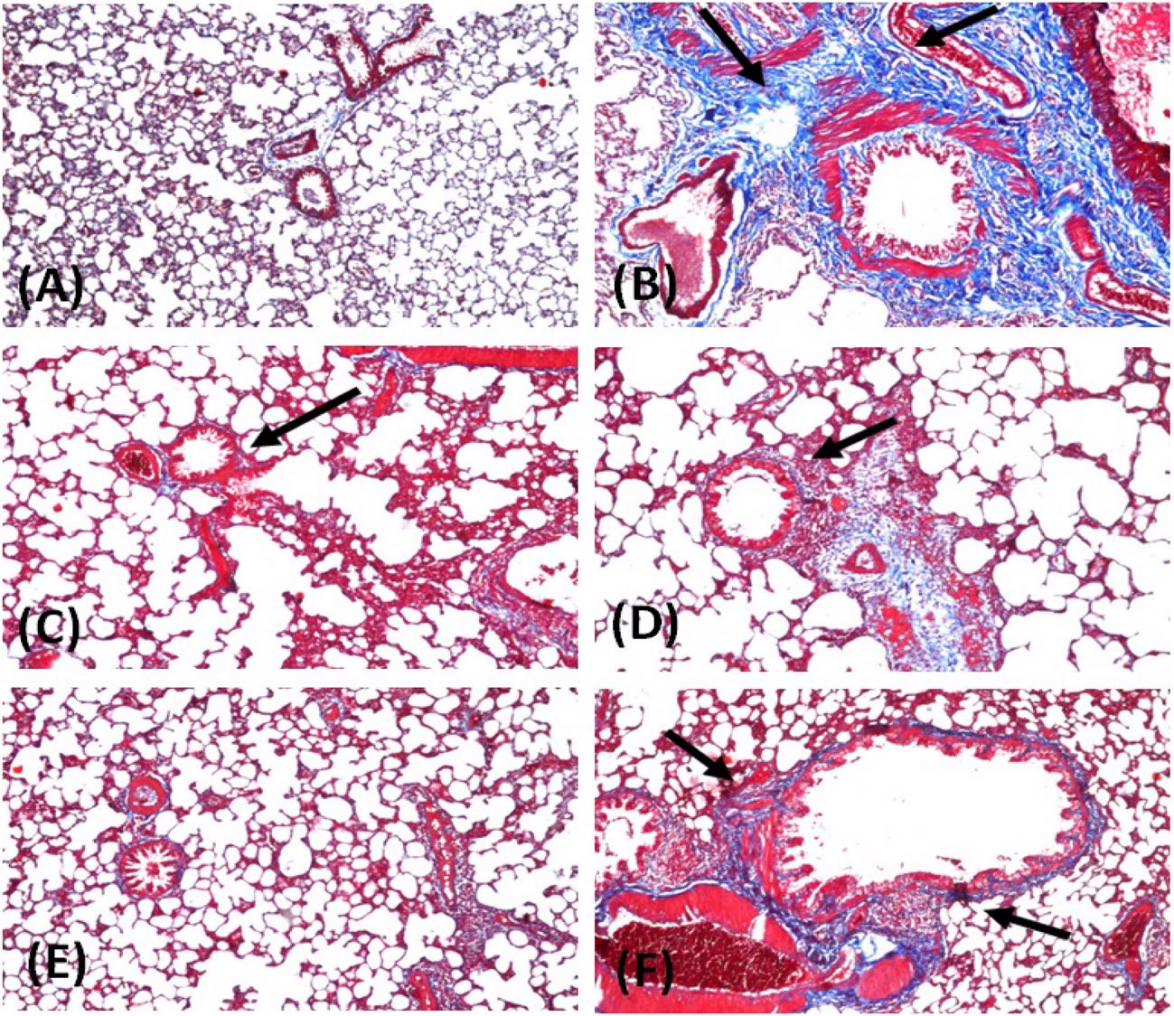
Photomicrographs of rat lung sections stained with Masson’s trichrome (X 16). (**A**) normal group (saline); (**B**) MTX-treated group; (**C**) Pir control treated group; (**D**) Pir + MTX treated group; (**E**) Cin control treated group; (**F**) Cin + MTX treated group using Masson’s trichrome stain

Severe proliferation of collagen and fibroblastic cells was revealed in the lung of MTX-received rats (Fig. 3B). Conversely, pre-treatment with either cin or pir in MTX-treated rats showed improvement with less appearance of fibrosis in the peribronchiolar and perivascular areas upon using Masson’s trichrome stain (Fig. 3D–F).

## Discussion

MTX is an antimetabolite drug used to treat types of cancer and rheumatoid arthritis through its antiproliferative properties (Juge et al. 2021). Several suggested pharmacological mechanisms of MTX action are suppression of thymidine and purine biosynthesis, inhibition of lymphoid tissue proliferation, particularly T lymphocytes, and initiation of the MAPK pathway, resulting in the motivation of activator protein-1 and nuclear factor-κB, which are fateful for inflammation regulation (Yan et al. 2021). Moreover, through the activation of oxidation systems, performed as an unneeded amendment in oxidative stress markers (MPO, MDA, and GSH), inflammatory cells penetrate the tissue, causing injury and cell death by generating DNA damage (Türk et al. 2022; Elsawy et al. 2021).

The present results revealed that oral administration of MTX produced lung oxidative stress alteration, as shown by a significant promotion in MPO activity as well as NO and MDA contents, versus a noticeable decline in lung content of total GSH. These outcomes are reliable in Al-Taher et al. (2020); Roghani et al. (2020); Sherif et al. (2020); and Jafaripour et al. (2021).

It was evident that MTX caused pulmonary toxicity due to its direct action via increased ROS production, leading to the activation of MPO, a lysosomal enzyme found in neutrophils responsible for hypochlorous acid production from hydrogen peroxide and chloride ions. The ROS production upturn, due to MTX, leads to NO and MDA intensification. NO induces the versatile oxidant peroxynitrite through a reaction with superoxide anions (Abouelela et al. 2019).

Moreover, the increase in MDA, the end product of polyunsaturated fatty acids, indicates lipid peroxidation (Roghani et al. 2020). While the oxidizing factors (MPO, MDA, and NO) were raised in lung tissue, the antioxidant factors (tGSH) were diminished during MTX administration, leading to an imbalance between the production of ROS and its elimination by antioxidant defenses (Mammadov et al. 2019; Ozcicek et al. 2020 and Mansour et al. 2021).

Additionally, the MTX-induced lung inflammation and fibrosis were revealed by a manifest escalation in the lung TNF-α, IL-8, TGF-β, and collagen contents. The significant elevation of inflammatory markers in addition to collagen as a fibrotic marker during MTX treatment was in harmony with Yamagami et al. (2020); Mohamed et al. (2021); Taskin et al. (2021) and Zaki et al. (2021). These actions could be attributed to the MTX’s ability to activate the leukocytes to increase the liberation of cytokines, which have a fundamental role in lung fibrosis formation through the epithelial-mesenchymal transition process and stimulation of NF-kβ and activator platelet-1 through ROS generation (Al Kury et al. 2020).

Conversely, the existing study demonstrated that the cin pre-treatment raised tGSH lung content, combined with a significant reduction in MPO activity as well as NO, MDA contents, TNF-α, IL-8, TGF-β, and collagen.

These observations are due to the antioxidant and anti-inflammatory properties of cin (Karatas et al. 2020; Babaeenezhad et al. 2021). It has been shown that cin derivatives suppress MAPK and AKT signaling pathways, causing NF-kβ, activator protein-1 attenuation (Abozaid et al. 2020; Godlewska-Żyłkiewicz et al. 2020; Hazafa et al. 2022). In agreement with our data, Abozaid et al. (2020) demonstrated the modulating effects of cinnamic acid on the redox signal and inflammatory response in an acute pancreatitis model, and Yan et al. (2018) referred to cinnamaldehyde's influence on IPF in mice via protesting against inflammation and oxidative stress. Besides, Ibrahim et al. (2020) discovered that cinnamic acid nanoparticles suppress apoptosis in an acute hepatitis rat model.

Pir was approved as the first therapy for mild and moderate IPF patients in the EU and USA as it has antioxidant, anti-inflammatory, and antifibrotic accomplishments (Tzouvelekis et al. 2017; Kreuter et al. 2021). Pir was taken in this study as a reference drug. Our results demonstrated that oral pre-treatment with pir enhanced the activity of tGSH. While reducing the activity of MPO, NO, and MDA contents, it also diminished TNF-α, IL-8, TGF-β, and collagen contents in the lung. The upshots match Ballester et al. (2020); Long et al. (2020) and Seifirad (2020).

In parallel, the histopathological examination showed that MTX caused a severe proliferation of collagen and fibroblastic cells, which indicates its fibrotic effect. Contrariwise, the effect of cin or pir pre-treatment mitigated the lung fibrosis caused by MTX administration.

Following pre-treatment with cin, our study revealed a notable reduction in MPO, NO, and MDA levels, along with an increase in tGSH level. This suggests that cin may hinder the activation of the JNK-signaling pathway while suppressing the effects of epidermal growth factor (EGF) and PDGF. Additionally, the decrease in TNF-alpha and IL-8, in conjunction with TGF-beta, may alleviate the activation of ERK and p38 mitogen-activated protein kinase signaling pathways. This would result in negative regulation of platelet aggregation and a decrease in the activation and proliferation of fibroblasts and collagen, indicating the advantageous impact of cin on MTX-induced lung fibrosis.

In conclusion, the present study revealed that pre-treatment with cinnamic acid caused a discernible escalation in tGSH content, combined with a significant diminution in MPO activity as well as NO and MDA contents, in addition to TNF-α, IL-8, TGF-β, and collagen. Optimistically, the data obtained from the present study showed a great resemblance between cin and pir, as the outcomes of cin administration were more or less the same as those of pir. Therefore, cin could be used as an adjuvant therapy with MTX, as it is a promising prophylactic treatment for lung fibrosis induced by MTX. The prophylactic effect of cinnamic acid against lung fibrosis is allied to its antioxidant, anti-inflammatory, and anti-fibrotic properties.

## Data Availability

The datasets generated during and/or analysed during the current study are available from the corresponding author on reasonable request.
